# Pacritinib inhibits proliferation of primary effusion lymphoma cells and production of viral interleukin-6 induced cytokines

**DOI:** 10.1038/s41598-024-54453-7

**Published:** 2024-02-19

**Authors:** Yiquan Wu, Victoria Wang, Robert Yarchoan

**Affiliations:** HIV and AIDS Malignancy Branch, Center for Cancer Research, National Cancer Institute, 10 Center Drive, Building 10, Rm. 6N106, MSC 1868, Bethesda, MD 20892-1868 USA

**Keywords:** Drug development, Virology, Lymphoma

## Abstract

Primary effusion lymphoma (PEL) and a form of multicentric Castleman’s disease (MCD) are both caused by Kaposi sarcoma herpesvirus (KSHV). There is a critical need for improved therapies for these disorders. The IL-6/JAK/STAT3 pathway plays an important role in the pathogenesis of both PEL and KSHV-MCD. We explored the potential of JAK inhibitors for use in PEL and KSHV-MCD, and found that pacritinib was superior to others in inhibiting the growth of PEL cell lines. Pacritinib induced apoptosis in PEL cells and inhibited STAT3 and NF-κB activity as evidenced by reduced amount of phosphorylated moieties. Pacritinib also inhibits FLT3, IRAK1, and ROS1; studies utilizing other inhibitors of these targets revealed that only FLT3 inhibitors exhibited similar cell growth inhibitory effects. FLT3’s likely contribution to pacritinib’s cell growth inhibition was further demonstrated by siRNA knockdown of FLT3. RNA sequencing and RT-PCR showed that many key host genes including cyclins and IL-6 were downregulated by pacritinib, while KSHV genes were variably altered. Finally, pacritinib suppressed KSHV viral IL-6-induced human IL-6 and IL-10 production in peripheral blood mononuclear cells, which may model an important step in KSHV-MCD pathogenesis. These results suggest that pacritinib warrants testing for the treatment of KSHV-MCD and PEL.

## Introduction

Kaposi sarcoma herpesvirus (KSHV) is a gammaherpesvirus that is the causal agent for several human malignancies and related disorders including Kaposi's sarcoma (KS), primary effusion lymphoma (PEL), KSHV-associated multicentric Castleman's disease (MCD), and KSHV inflammatory cytokine syndrome (KICS)^1–4^. These conditions most frequently arise in individuals co-infected with human immunodeficiency virus (HIV). PEL is a lymphoma that tends to occur in body effusions; essentially all PEL tumors are infected by KSHV, while a substantial majority are co-infected by Epstein-Barr virus (EBV)^5,6^. KSHV-MCD is a lymphoproliferative disorder pathologically characterized by KSHV-infected plasmablasts in the germinal center of lymph nodes. Patients have severe inflammatory symptoms caused by an excess of inflammatory cytokines. There is increasing appreciation that PEL and KSHV-MCD are often underdiagnosed and in fact KSHV-MCD has recently been reported to represent 16% of lymphoproliferative disease in HIV-infected individuals in sub-Saharan Africa^7^. PEL and KSHV-MCD are both fatal if not treated, and even with current therapies, PEL still carries a substantial mortality rate^8^. KSHV-MCD often responds to rituximab, but relapses are common^9,10^. Therapy of all these is complicated by the fact that people living with HIV and KSHV infection often develop multiple KSHV-related diseases simultaneously. Thus, the management of PEL and KSHV-MCD remains challenging, and there is a high unmet need for effective and well-tolerated treatments.

There is evidence that interleukin-6 (IL-6), interleukin-10 (IL-10), and KSHV-encoded viral IL-6 (vIL-6), play important roles in the pathogenesis of PEL and of KSHV-MCD. IL-6 binds to IL-6 receptor α and gp130, and then signals through the Janus kinase (JAK) / signal transducer and activator of transcription (STAT) pathway^11,12^. KSHV vIL-6 physically interacts with gp130 independent of the gp80 IL-6R subunit, and can then thus activate JAK/STAT signaling through gp130 alone^13–16^. Through this mechanism, vIL-6 can promotes proliferation in PEL cells. Because of its direct binding to gp130, it is also more promiscuous in the cells it can activate^13–16^. Also, vIL-6 can bind to gp130 intracellularly on the endoplasmic reticulum^17^. With regard to PEL, IL-6 and IL-10 have both been shown to be autocrine factors. Also, inhibition of STAT3 has been shown to be toxic to PEL cells in vitro^18^. With regard to KSHV-MCD, there is evidence that inflammatory flares are caused by an excess of serum IL-6, IL-10, and vIL-6^19,20^. In a humanized mouse model of KSHV-MCD in which vIL-6 induces clinical disease manifestations, clinical disease does not develop if human IL-6 is knocked out^21^. Thus, there is evidence that the pathogenesis of KSHV-MCD involves the stimulation of IL-6 (and potentially IL-10-producing cells) by vIL-6. The available evidence thus suggests that these cytokines and the signaling induced by them are targets worth exploring as therapeutic targets in these diseases.

Given that human IL-6 and vIL-6 (as well as IL-10) signal through gp130 and the JAK/STAT pathway and that STAT3 has been shown to be toxic to PEL cells in vitro^18,22,23^, we decided to explore JAK inhibitors as possible therapy for PEL and KSHV-MCD. JAKs are a family of intracellular enzymes involved in regulating cell signaling pathways (For review, see Ref.^23^). In addition to playing a crucial role in the signaling from the IL-6 receptor, they are also involved in the signaling through other cellular receptors. The therapeutic potential of JAK inhibitors has been recognized for several decades, and several drugs in this class have recently been developed and approved for the treatment of various conditions^24,25^. With this background, we investigated the potential utility of JAK inhibitors in the treatment of PEL and KSHV-MCD.

## Results

### JAK inhibitors variably affect PEL cell growth

**Figure 1:**
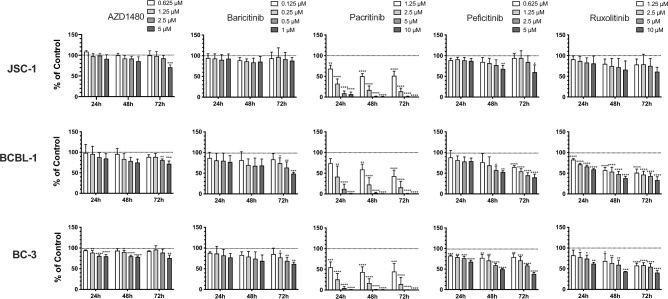
JAK inhibitors variably affect PEL cells growth. JSC-1, BCBL-1, and BC-3 cells were treated in triplicate with indicated concentrations of AZ1480, baricitinib, pacritinib (Selleck Chemicals LLC), peficitinib or ruxolitinib, or with DMSO control for up to 72h. The number of viable cells was assessed at 24 h, 48 h and 72 h using CellTiter-Glo Luminescent Cell Viability Assay. Shown are mean relative light units (RLU) from three independent experiments. Error bars indicate the standard deviations. Asterisks indicate p values: *p < 0.05, **p < 0.01, ***p < 0.001, ****p < 0.0001.

There are no KSHV-MCD cell lines, and because JAK/STAT signaling has been shown to be important for the survival of PEL cells^18^, we tested the activity of various JAK inhibitors against PEL cell lines. In initial experiments, we compared the activities of five JAK inhibitors (AZ1480, baricitinib, pacritinib, peficitinib and ruxolitinib) on the in vitro growth of PEL cell lines. Drug concentrations tested were based on the plasma serum C_max_ concentrations (Supplementary Fig. S2)^26–30^ determined in patients, with the understanding that because of variations in protein binding between human plasma and 15% FCS of these compounds, there may not be a one-to-one correlation in the free drug. The concentrations utilized ranged from about one quarter of the C_max_ to slightly above the C_max_. Serial dilutions of each inhibitor were applied to the PEL cell lines including JSC-1, BCBL-1 and BC-3 for up to 48 h (Fig. 1).

By comparison with the other 4 inhibitors, pacritinib exhibited relatively superior cytotoxicity. We then tested pacritnib’s effect on 2 other PEL cell lines including BC-1 and BC-2, and 2 lymphoma lines without KSHV including BJAB and CA46. BC-1 and BC-2 showed similar time- and dose-dependent growth inhibition as compared to the initial lines tested (Supplementary Fig. S3C and S3D). The virus-negative lymphoma lines BJAB and CA46 also showed some inhibition with pacritinib, but less than the PEL lines (Supplementary Fig. S3A and S3B). Since we had two sources of pacritinib, we compared their activities on PEL cells and both pacritinib from CTI BioPharma Corp. and from Selleck Chemicals LLC exhibited consistent growth inhibition effect on JSC-1 and BCBL-1 cells (Supplementary Fig. S4).

**Figure 2:**
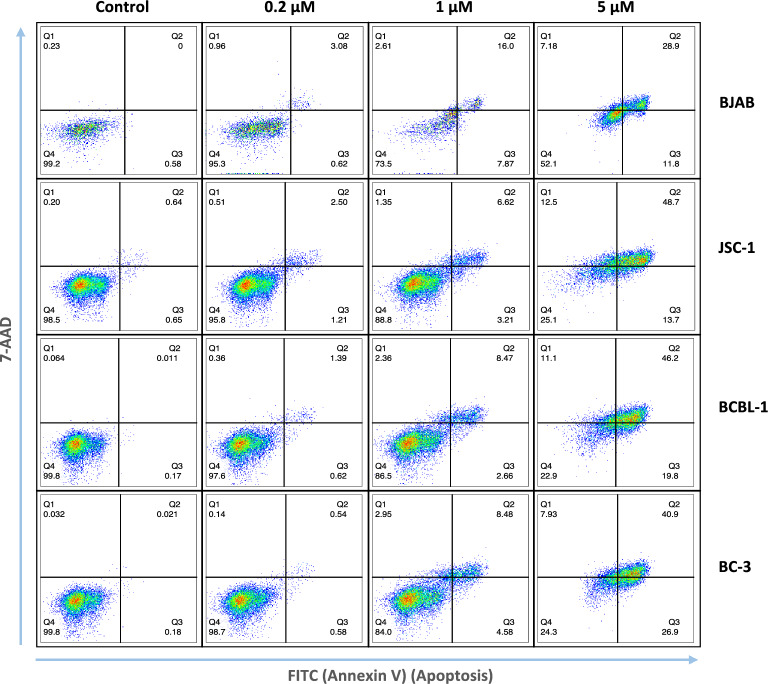
Pacritinib induces apoptosis and cell death on cell lines with or without KSHV. BJAB, JSC-1, BCBL-1, and BC-3 cells were treated for 24 h with 0.2 μM, 1 μM, 5 μM pacritinib (Selleck Chemicals LLC) or DMSO control (Control), then stained with FITC Annexin V Apoptosis Detection Kit with 7-AAD. Cell apoptosis (Annexin V) and cell death (7-AAD) were analyzed by flow cytometry. Q1: 7-AAD along stained cells, Q2: 7-AAD and Annexin V-FITC stained cells, Q3: Annexin V-FITC stained cells, Q4: cells not stained.

To explore the mechanism for the growth inhibition, we treated a B cell line without KSHV BJAB, and PEL lines including JSC-1, BCBL-1 and BC-3, with increasing amounts of pacritinib for 24 h, and then evaluated cell apoptosis and cell death using flow cytometry. As seen, pacritinib induced cell apoptosis and cell death in all the 4 cell lines in a dose-dependent manner (Fig. 2, supplementary Fig. S5).

### Pacritinib downregulates JAK2, STAT3 and NF-κB activities

**Figure 3:**
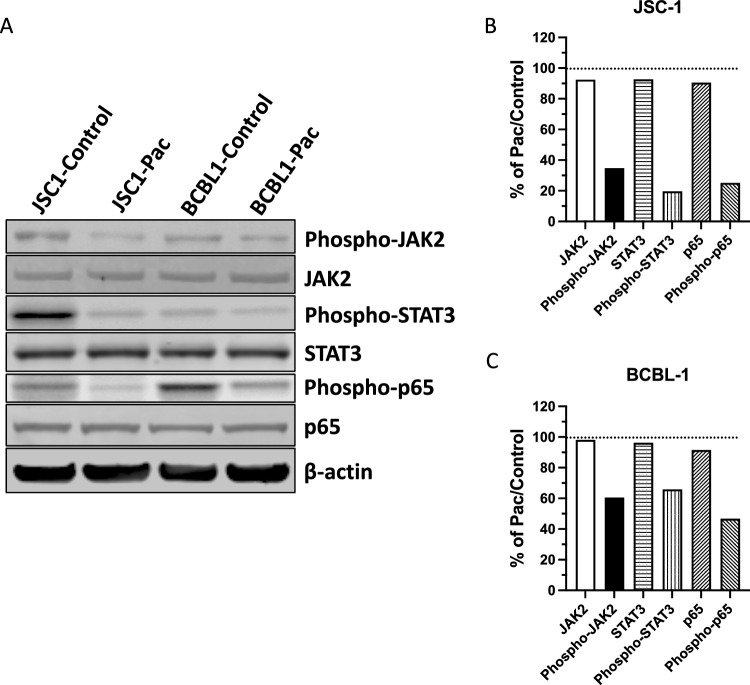
Pacritinib inhibits activities of JAK2, STAT3 and NF-κB. Protein extracts of JSC-1 and BCBL-1 cells treated for 48 h with 1 μM pacritinib (CTI BioPharma Corp.) or DMSO control were analyzed for the total and phosphorylated JAK2, STAT3 and p65 of NF-κB by Western blot. (**A**) JAK2, STAT3 and p65 indicate the total amount of the protein; pJAK2, pSTAT3 and p65 indicate the phosphorylated forms of the proteins, and β-actin serves as the internal control. Intensity of each band in JSC-1 group. Western blots were stripped after the initial probe of the phosphorylated proteins and β-actin and then probed with antibodies to the unphosphorylated proteins. The full scanned Western blots of Fig. 3A are provided in Supplemental File 12. (**B**) Signal intensities for JSC-1 and (**C**) BCBL-1 cells were normalized according to β-actin, and signal of all total and phosphorylated JAK2, STAT3 and p65 of NF-κB in the Pac-treated cells were presented as percentages compared with that of the control cells.

The gp130 receptor for IL-6 and other cytokines signal through the JAKs and STAT3 to enhance the activation of NF-κB. In addition to JAK2, another direct target of pacritinib, IRAK-1, also activate NF-κB. Here we wanted to determine if exposure of PEL cells to pacritinib would affect these signaling proteins and ultimately their downstream targets such as NF-κB. A number of KSHV genes, such as KSHV-encoded FLICE inhibitory protein (vFLIP) can affect NF-κB without involving gp130, and it was not clear that these targets would in fact be affected by pacritinib treatment of PEL cells. To explore this, we assessed the effect of pacritinib treatment on JAK2, STAT3 phosphorylation and NF-κB activation. As shown in Fig. 3, both JSC-1 and BCBL-1.

PEL cells exposed to pacritinib had a decrease in phosphorylated JAK2 and STAT3 as well as a decrease in pp65 (one of the main components of the NF-κB complex). This provides evidence that pacritinib was in fact affecting these factors that were downstream of JAK, and this result was consistent with the previously reported finding that inhibition of STAT3 was toxic to PEL cells. However, it was possible that the effects were being mediated in part by effects of pacritinib on other kinases.

### FLT3, beyond JAK2, contributes to the effects of pacritinib in PEL cells

**Figure 4:**
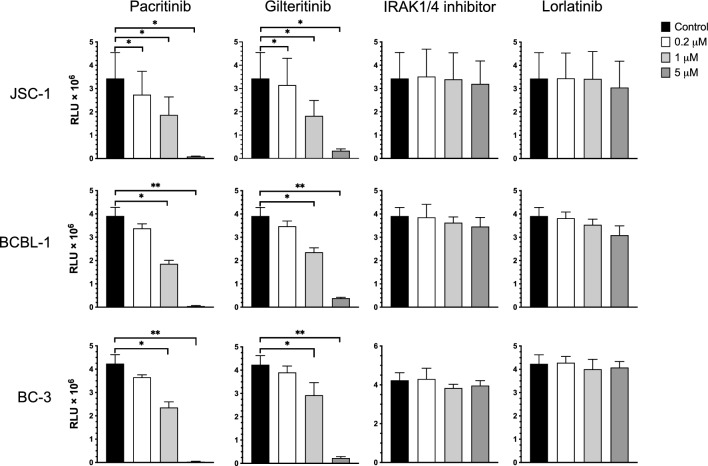
Effects of various kinase inhibitors on the growth of PEL cell lines. JSC-1, BCBL-1, and BC-3 cells were treated in triplicate for 72 h with 0.2 μM, 1 μM or 5 μM of pacritinib (Selleck Chemicals LLC), gilteritinib (FLT3 inhibitor), IRAK1/4 inhibitor or lorlatinib (ROS1 inhibitor), or with DMSO medium control. The number of viable cells was assessed using CellTiter-Glo Luminescent Cell Viability Assay. Shown are relative light units (RLU) from three independent experiments. Error bars indicate the standard deviations. Asterisks indicate p values: *p < 0.05, **p < 0.01, ***p < 0.001, ****p < 0.0001. Those without asterisks are not significant (p > 0.05).

Several studies have provided evidence that IL-6 signals predominantly through JAK1 rather than JAK2^23,24^, and given this, we were somewhat surprised that pacritinib had relatively better activity against PEL cells than the other tested JAK inhibitors, several of which targeted both JAK1 and JAK2. There is evidence that pacritinib also targets certain other kinases, including Fms-related receptor tyrosine kinase 3 (FLT3), interleukin-1 receptor-associated kinase-1 (IRAK1), and ros proto oncogene 1 (ROS1), at relatively low concentrations^28^, and we wondered if its activity against PEL might be in part due to its effects on these other kinases. To explore this, we assessed the cytotoxicity of PEL cell lines treated with various concentrations of pacritinib or inhibitors targeting these other kinases including gilteritinib (FLT3), IRAK1/4 inhibitor, and lorlatinib (ROS1). For all the tested PEL cell lines including JSC-1, BCBL-1 and BC-3, IRAK1/4 inhibitor and lorlatinib had little impact on cell growth at the doses tested. By contrast, gilteritinib showed growth inhibition at 1 µM and 5 µM (Fig. 4).

To explore this further, we tested several other small molecule FLT3 inhibitors (FF10101, G-749, MRX-2843, and quizartinib) (Supplementary Fig. S6). All these inhibitors similarly inhibited the growth of both JSC-1 and BCBL-1 cells at concentrations of 1 µM or 5 µM. Taken together, these results suggested that the activity of pacritinib on PEL cells was at least in part mediated by its inhibition of FLT3.

**Figure 5:**
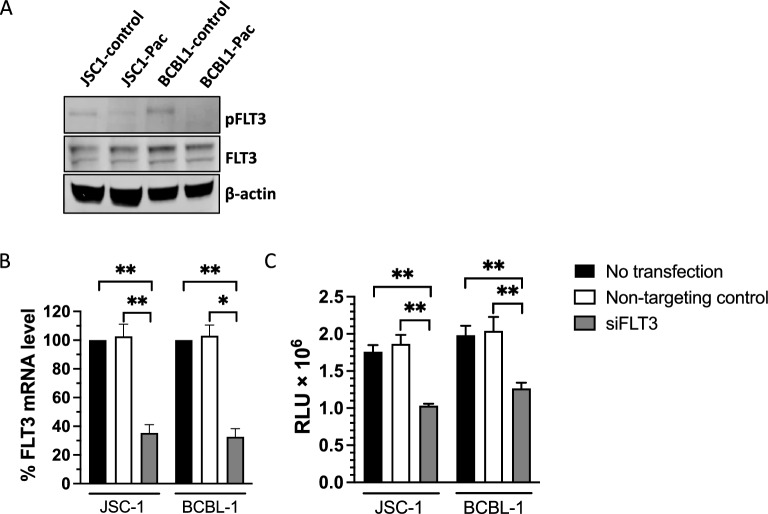
FLT3 contributes to pacritinib’s cytotoxicity effect in PEL cell lines. (**A**) Levels of FLT3 and phosphorylated FLT3 (pFLT3) in protein extracts of JSC-1 and BCBL-1 cells treated for 72 h with 1 μM pacritinib (Pac) or DMSO control. Extracts were analyzed for the activities of pFLT3 and (after stripping) FLT3 by Western blot. β-actin serves as the internal control. Cells were transfected with siRNA target FLT3, non-target control, and no transfection control. Expression of FLT3 mRNA was tested by qPCR. The full scanned Western blots are provided in Supplemental Fig. 12. (**B**) The number of viable cells (**C**) was assessed using CellTiter-Glo Luminescent Cell Viability Assay. Shown are the data from 3 independent experiments. Error bars indicate the standard deviations. Asterisks indicate p values: **p < 0.01, ***p < 0.001, ****p < 0.0001.

To further assess the possible role of FLT3 inhibition on the toxicity of pacritinib against PEL, we tested the FLT3 activity in pacritinib-exposed PEL cells by Western blot, and found that pacritinib reduced phosphorylated FLT3 in both JSC-1 and BCBL-1 cells (Fig. 5A). We then used siRNA to knock down FLT3 expression in JSC-1 and BCBL-1 cells and verified that there was in fact a decrease in FLT3 mRNA (Fig. 5B). Moreover, siRNA induced a significant decrease in the number of viable cells as measured by ATP-based CellTiter-Glo^®^ Luminescent Cell Viability Assay, whereas a non-targeting control showed no decrease (Fig. 5C). All these data provide evidence inhibition of FLT3 activity contributes to its growth inhibition effect in PEL cells.

We next assessed the relative contributions of JAK2 and FLT3 to pacritinib’s inhibitory effect on PEL cell growth. JSC-1 and BCBL-1 cell lines were exposed to varying concentrations of pacritinib, gilteritinib (an FLT3 inhibitor), AZD1480 (a JAK2 inhibitor), or a combination of gilteritinib and AZD1480. As previously seen in Fig. 4, gilteritinib had substantial activity against PEL (Supplementary Fig. 7). In addition, AZD1480 also reduced the growth of PEL cells, and the combination of gilteritinib and AZD1480 was more potent than gilteritinib alone (Supplementary Fig. 7C and D). Taken together, these results suggest that both JAK2 and FLT3 inhibition contribute to the activity of pacritinib against PEL.

### Pacritinib reshapes cellular and viral gene expression profiles

**Figure 6:**
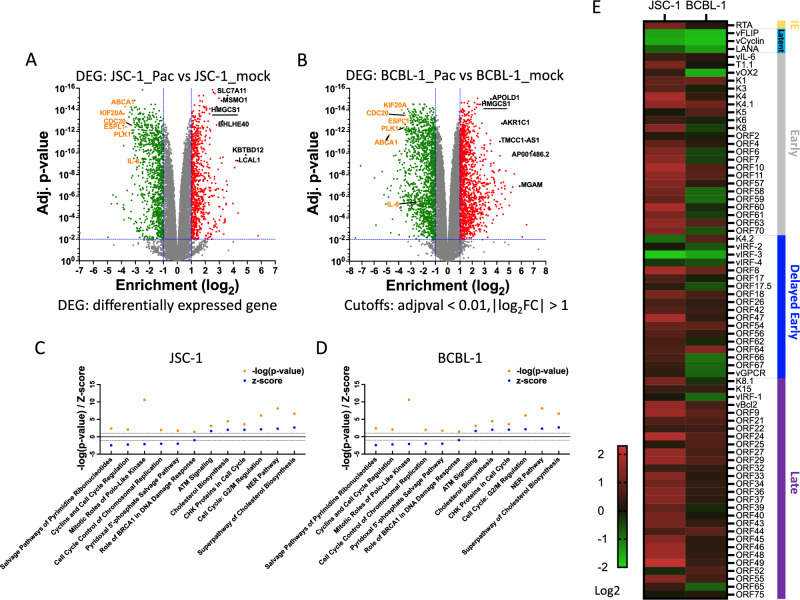
Pacritinib reshapes cellular and viral gene expression profiles. RNA extracted from JSC-1 and BCBL-1 cells treated for 24 h with 0.5 μM pacritinib or DMSO control was subjected to RNA-Seq for mRNA expression analysis of both human and KSHV genes. ((**A**) and (**B**)) Sequences were aligned to human genome. Volcano plots summarizing the deregulated human genes in the two groups. The log_2_ enrichment indicates the mean fold change for each gene. Each dot represents one gene. Utilizing a cutoff with the adjusted p value (adjpval) < 0.01, and the absolute value of log_2_FC > 1, grey dots represent differentially expressed genes (DEG)s without significant change between control group and treated group, green dots represent down-regulated genes and red dots represent up-regulated genes. Some genes whose expression is substantially altered are labeled with their names; down-regulated genes are colored in orange and up-regulated ones in black. ((**C**) and (**D**)) Canonical pathway analysis of the DEGs from JSC-1 group (**C**) and BCBL-1 group (**D**) acquired from IPA. Shown are enriched pathways with cutoff of |Z-score|≥ 1 and p-value p < 0.05. (**E**) Sequences were aligned to KSHV genome. Fold change of KSHV gene expression is shown in heatmap in log2 scale. Genes are classified according to their expression in latent or stages of lytic replication.

To further explore the mechanism for the effects of pacritinib on the inhibition of PEL cell line growth, we assessed the cellular and viral gene expression profile in response to pacritinib treatment. To this end, we used deep RNA-Seq technology to assess the mRNA in JSC-1 and BCBL-1 cells treated with 1 μM pacritinib for 24 h and compared the results to untreated cells (Fig. 6A and B). Both JSC-1 and BCBL-1 PEL cells exhibited significant changes in their cellular gene expression profile with a substantial number of genes being either upregulated or downregulated. Among these differentially expressed genes (DEGs) with a more than two-fold change in expression and p < 0.01, there were 474 that were downregulated and 711 upregulated genes in both PEL cell lines (Supplementary Fig. S8A). Interestingly, among the most significant DEGs (FC > 8, p < 0.0001), there were more^28^ co-downregulated genes, but less significant upregulated genes^4^ showed up in both groups (Supplementary Fig. S8B). To further investigate how pacritinib reshaped the cellular pathways, we performed enrichment analysis of these DEGs and found that several pathways relevant to cell cycle and proliferation were inhibited in both cell lines (Fig. 6C,D and Supplementary Fig. S9). Notably, in addition to DEGs related to cyclins and cell cycle regulation, IL-6 was also downregulated by sevenfold to 14.28% in JSC-1 group while downregulated by over threefold to 31.53% in BCBL-1 group (Fig. 6A and B). Expression of several DEGs including co-upregulated HMGCR and co-downregulated IL-6, CDK1 and PLK1 was further confirmed by qPCR (Supplementary Fig. S10A). There was some discordance in the expression of HMGCR, which was upregulated by RNA-Seq in both JSC-1 (FC = 2.32) and BCBL-1 cells (FC = 2.55), but only minimally upregulated or unchanged by qPCR. Other than this, there was good concordance between these two techniques.

We also examined the effects of pacritinib treatment on the expression of KSHV genes. By RNA Seq, the KSHV latent genes were generally downregulated by pacritinib in both JSC-1 and BCBL-1 although there was some variability (Fig. 6E). Some of the lytic genes were downregulated in BCBL-1, while pacritinib upregulated most of the lytic genes of JSC-1 (Fig. 6E). We further confirmed the viral gene expression changes of select genes using qPCR and found general concordance with the results of RNA Seq, although there was no significant change in LANA expression in JSC-1, compared to a slight decrease by RNA-Seq. Interestingly, there was no significant effect of pacritinib on vIL-6 mRNA expression in either BCBL-1 or JSC-1 (Supplementary Fig. S10B). Next, we assessed whether pacritinib induces the production of KSHV virions in the medium. Virus pelleted from the supernatant of treated JSC-1 and BCBL-1 was assessed by assaying for ORF45 protein, an abundant component within KSHV virions, by Western blot^31^. In the absence of 12-O-tetradecanoylphorbol-13-acetate (TPA), a lytic inducer, or pacritinib, there was essentially no detectable virus in the supernatant, while exposure to TPA induced strong production of KSHV in both cell lines. Pacritinib induced a low level expression of virus in JSC-1 cells, but not BCBL-1 cells (Supplementary Fig. S11).

### Pacritinib blocks vIL-6- or LPS-induced IL-6 and IL-10 production in PBMC

**Figure 7:**
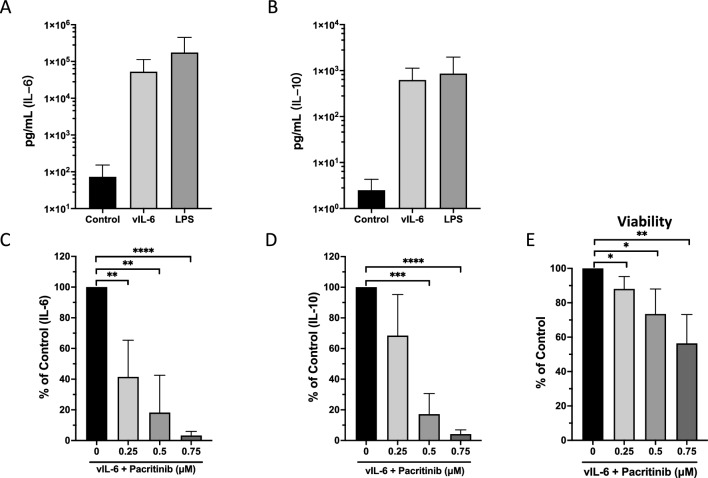
Pacritinib blocks vIL-6- or LPS-Induced IL-6 and IL-10 production in PBMC. PBMC were treated with 1 μg vIL-6-MBP or 1 μg LPS, and then with indicated amounts of pacritinib or media control for 48 h. Supernatants were then collected and analyzed for IL-6 ((**A**) and (**C**)) and IL-10 ((**B**) and (**D**)) expression by ELISA. The number of viable cells was assessed using CellTiter-Glo Luminescent Cell Viability Assay (**E**). Data are all normalized to the control groups with 0 μM pacritinib. Shown is the data from five independent experiments. Error bars indicate the standard deviations. Asterisks indicate p values: *p < 0.05, **p < 0.01, ***p < 0.001, ****p < 0.0001. Those without asterisks are not significant (p > 0.05).

A hallmark of KSHV-MCD and KICS is elevated levels of IL-6 and IL-10 in the patients. KSHV-encoded vIL-6 signals through the JAK/STAT pathway and STAT1 and STAT3 activation^13^, and can thus potentially upregulates IL-6 and IL-10 production. Since we found that pacritinib inhibited the activities of STAT3 and NF-κB in PEL cells, we explored pacritinib’s ability in blocking of vIL-6-induced production of IL-6 and IL-10 in an ex vivo setting. PBMC were isolated from the different donors, and treated with increasing amounts of pacritinib and 1 μg vIL-6 or 1 μg LPS, and supernatants were taken for the measurement of IL-6 and IL-10. PBMC released very low basal levels of IL-6 (mean of 72.8 pg/mL) and IL-10 (mean of 2.5 pg/mL) in the supernatant. Both vIL-6 and LPS stimulated IL-6 to 720 and 2394 times higher (Fig. 7A), and IL-10 to 622 and 862 times higher (Fig. 7B) separately. Although, pacritinib had dose-dependent cytotoxicity in PBMC, it showed strong inhibition of both IL-6 and hIL-10 production. For example, there were about 50% of live cells (Fig. 7E) left but only 1.7% of IL-6 (from an average of 52,482 pg/ml to 905 pg/ml) and 3.9% of IL-10 (from an average of 622 pg/ml to 25 pg/ml) left when treated with 0.75 μM pacritinib (Fig. 7C and D) for two days. These observations fit to the result that pacritinib-treated cells had reduced activity of STAT3 and NF-κB.

## Discussion

In this manuscript, we explore the activity of a number of JAK inhibitors against PEL and show that, pacritinib, which is approved in the United States for the treatment of patients with myelofibrosis and severe thrombocytopenia^32^, is particularly active and induces both apoptosis and necrosis. By contrast, several other inhibitors tested with activity against JAK1 or both JAK1 and JAK2 had relatively little activity against PEL. This was somewhat surprising, as pacritinib is relatively specific for JAK2 and has little activity against JAK1, and there is evidence that gp130 signals predominantly through JAK1^24,33^. These results suggested that while inhibition of JAK2 may have contributed to the activity of pacritinib against PEL, this was not the sole mechanism.

It has become evident that pacritinib also has activity against several other kinases such as ROS1, IRAK1, and FLT3 with IC50s reported in lower nanomolar ranges^28^. We explored the possibility that activity against one or more of these other kinases might contribute, or even be the main mediator, of its activity against PEL. We observed that gilteritinib, a FLT3 inhibitor, also suppressed PEL growth, while small molecule inhibitors of IRAK1/4 and ROS1 did not. Moreover, we found that PEL cells contained phosphorylated FLT3 and that the level of phosphorylated FLT3 was decreased by pacritinib. Finally, we found that siRNA against FLT3 decreased the growth of PEL cells. FLT3 activates pathways inhibiting apoptosis, while promoting proliferation^34^. Mutations of this kinase are frequently associated with acute myelogenous leukemia, and FLT3 inhibitors can be used to treat this disease. This somewhat unexpected result suggests that gilteritinib or other FLT3 inhibitors may be worth exploring further in PEL.

Analysis of changes in RNA expression in PEL induced by pacritinib revealed a number of genes whose activity were decreased or increased. FLT3 signals through a number of factors, including AKT, ERK, and STAT5, to ultimately affect cell proliferation and survival^35^. Consistent with this, pathway analysis here showed a number of dysregulated pathways, including several related to the cell cycle. FLT3 is involved in the development of early hematopoietic progenitor cells^36^. However, FLT3 is also expressed on developing B cells, appears on peripheral B cells after activation^37^, and has been shown to be overexpressed in multiple myeloma^38^, a disease that bears a number of similarities to PEL with regard to the state of B cell differentiation. Thus, it is plausible that inhibition of FLT3 would suppress the growth of PEL cells. At the same time, given the activity of another JAK2 inhibitor (AZD1480) alone and in combination with pacritinib and previous studies showing the importance of JAK/STAT signaling in PEL^18,39^, the data suggest that JAK2 inhibition contributes to the PEL activity. In a similar manner, pacritinib has been shown to have activity in acute myeloid leukemia cells with drug resistance to FLT3 therapy because of its combined JAK2/FLT3 activity^40^.

Yang et al. in the Dittmer lab, have previously reported that IRAK1 is a driver mutation for PEL^41^, and in a follow-up paper by Seltzer from that laboratory, it was reported that IRAK1/4 inhibitor suppressed the growth of PEL cells^42^. We did not find inhibition of PEL cells by IRAK1/4 inhibitor; however, our results with this inhibitor are not inconsistent with those of Seltzer et al., in that they only saw inhibition at substantially higher concentrations (> 20 µM) than tested here. Also, additional CRISPR/Cas9 studies by the Dittmer group showed that PEL cells could circumvent IRAK1 and IRAK4 loss, calling into question the potential of IRAK1/4 inhibition by itself as a treatment for PEL. Nonetheless, it remains possible that IRAK1/4 inhibition may contribute to the activity of pacritinib in PEL.

In addition to its effect on cellular genes, we found that pacritinib could suppress the expression of some KSHV genes and especially those involved in latency. The mechanism for these effects is unclear, but are likely at least in part secondary to the effects on cellular kinases. KSHV latent genes can contribute to the PEL oncogenesis through a variety of mechanisms, including inhibition of apoptosis, and inhibition of tumor suppressor genes. Moreover, KSHV-encoded vFLIP can activate cellular proliferation and induce the expression of IL-6 in part through its effects on NF-κB^43^. Thus, it is likely that the suppression of these KSHV genes also contribute to the suppression of PEL growth by pacritinib.

vIL-6 and IL-6 signaling are also very important in the pathogenesis of KSHV-MCD, and we also explored the potential utility of pacritinib in this condition. In a humanized mouse model of KSHV-MCD, vIL-6 can induce an MCD-like condition; however, knocking out the human IL-6 in this model abrogates the MCD-like disease^21^. Monoclonal antibodies to IL-6 or the IL-6 receptor have been shown to be effective in classical MCD, which is largely mediated by IL-6^44^. However, we found only modest activity of tocilizumab, an antibody to the IL-6 receptor, in patients with KSHV-MCD^45^; this may be because it did not block vIL-6 activation through gp130 and the resulting induction of IL-6, IL-10, and other cytokines. We hypothesized that an inhibitor of gp130 signaling through JAK inhibition might be more effective, and in this study have found that pacritinib could effectively block vIL-6-induced hIL-6 and hIL-10 production by PBMC. This is consistent with other studies showing that pacritinib can inhibit NF-κB phosphorylation and production of IL-6^46–48^, perhaps through a combination of its activity against JAK2 as well as other kinases (such as IRAK1). Whatever the mechanism, these results suggest that pacritinib may be worth exploring as a treatment for KSHV-MCD.

Pacritinib is now approved in the United States for the treatment of patients with myelofibrosis who have severe thrombocytopenia, and it is relatively well tolerated; the main toxicities are diarrhea, thrombocytopenia, nausea, and anemia and they are reported to be easily managed^32^. The results here provide evidence that pacritinib can block the growth of PEL cells in vitro and also vIL-6-induced human IL-6 and IL-10 production. Taken together, these results support the exploration of pacritinib as a possible therapy for KSHV-MCD and/or PEL, and our group is in the process of initiating a trial to test this possibility.

## Methods

### Cells and cell culture

Two uninfected Burkitt lymphoma B-cell lines, BJAB and CA46, and three PEL cell lines, BC-1, BC2 and BC-3 were acquired from American Type Culture Collection (ATCC), Manassas, VA. The PEL cell line BCBL-1 was obtained from the National Institutes of Health AIDS Research and Reagent Program, Rockville, MD. The PEL cell line JSC-1 line was a gift from Richard Ambinder, John Hopkins University, Baltimore, MD. JSC-1, BC-1, and BC-2 cells are co-infected with EBV, while BCBL-1 and BC-3 are not. All cells were cultured in RPMI 1640 (Life Technologies) with 15% heat-inactivated fetal calf serum (FCS) (Hyclone), 100 units/mL penicillin, 100 μg/mL streptomycin sulfate and maintained at 37℃ and 5% CO_2_ in Falcon cell culture flasks. For the treatment of cells with inhibitors, floating cells were seeded at 2 × 10^5^/mL and treated with various inhibitors at indicated concentrations for up to 3 days.

### Test compounds

JAK Inhibitors (AZD1480, baricitinib, peficitinib, ruxolitinib and pacritinib), Fms-related receptor tyrosine kinase 3 (FLT3) inhibitors (gilteritinib, quizartinib, FF10101, G-749 and MRX-2843) and other kinase inhibitors including an inhibitor of interleukin 1 receptor associated kinase 1 (IRAK1) and IRAK4, (IRAK1/4 inhibitor), and the ros proto oncogene (ROS1) /anaplastic lymphoma kinase (ALK) inhibitor lorlatinib were purchased from Selleck Chemicals LLC. Pacritinib was also provided by CTI BioPharma Corp. through a Materials Collaborative Research and Development Agreement (M-CRADA). Inhibitors were dissolved in DMSO at a stock concentration of 10 mM/mL, except for pacritinib at 5 mM/mL. All stocks were aliquoted and stored at − 20 °C.

### Assays for viable cells

The relative number of viable cells was analyzed using CellTiter-Glo^®^ Luminescent Cell Viability Assay kit (Promega, Cat. No. G7571). Briefly, 50 μL CellTiter-Glo^®^ reagent was added to 50 μL cells in 96-well plate. Contents were mixed for 2 min on an orbital shaker and then incubated at room temperature for 10 min. Luminescence was recorded using a plate reader VICTOR™ X3 (PerkinElmer). The percentage of alive vs dead cells was assessed using trypan blue staining.

### Flow cytometry analysis

Cell death was assessed by FITC Annexin V Apoptosis Detection Kit with 7-AAD (Cat. #:640,922) from BioLegend according to the manufacturer’s instructions and then analyzed with a flow cytometry calibur™ Flow Cytometry system (BD Biosciences). Data were analyzed and visualized using FlowJo software (flowjo.com).

### RNA sequencing

Total RNA was extracted using Direct-Zol Miniprep kit (Zymo), following manufacturer’s instructions. Library preparation and sequencing was performed by NCI CCR Sequencing facility. Ribosomal RNA was removed, and sequencing libraries were prepared using Illumina TruSeq Stranded / NEBnext Ultra Low Input Total RNA Library Prep and paired-end sequencing. The reads from the FASTQ files (reads were generated by HiSeq4000) were trimmed of Illumina adapters using the Fastx toolkit. The reads were aligned to two target genomes, human (GRCh38) and KSHV (Genbank accession number NC_009333.1) using TopHat^49^. Transcripts were assembled using Cufflinks and Cuffdiff^50^ in order to reveal differentially expressed genes. Significant mRNA fold change was determined by an adjusted p-value ≤ 0.05 based on the Benjamini and Hochberg multiple testing correction. DEGs from JSC-1 group and BCBL-1 group were determined by fold change ≥ 2, adjusted p-value < 0.05. Pathway enrichment analysis was performed with Ingenuity Pathway Analysis (IPA, Qiagen). Enriched pathways with p < 0.05 and |Z-score|≥ 1 were selected and presented using GraphPad Prism 9. GraphPad Prism 9 was also used for visualization of heat maps and scatter plots.

### siRNA knockdown

Accell siRNA delivery media (Cat. No. B-005000-500), Accell Non-targeting Pool siRNA (negative control, Cat. No. D-001910-10-05), Accell GAPDH Pool siRNA (positive control, Cat. No. D-001930-10-05), and siRNA pool targeting FLT3 (Cat. No. E-003137-00-0005) were obtained from Dharmacon/Horizon Discovery. Briefly centrifuge siRNA down in the tubes and resuspend with RNase-free water to a 100 µM stock concentration. JSC-1 or BCBL-1 cells were plated at 2 × 10^5^/mL in 1/well mL RPMI 1640 with 5% FCS the day before in a 24-well plate, and washed once with 1 mL Accell siRNA delivery media the next morning. Then cells were resuspended in 1 mL Accell delivery mix which contains 990 μL Accell delivery media and 10 μL of each 100 μM stock siRNA pool. The final concentration will be 1 μM Accell siRNA per well. Incubate cells at 37 °C with 5% CO_2_ for 24 h. Then 20 μL/well FBS was added to each well, and incubated for another 48 h. Assessment of knockdown of mRNA was accessed using qPCR. Viability of the cells was further evaluated using CellTiter-Glo^®^ Luminescent Cell Viability Assay as described above.

### RT-qPCR

mRNA was extracted using Direct-zol RNA Miniprep Kit (Zymo Research, Cat. No. R2052) following the instruction. cDNA synthesis was performed using random primers with the High-Capacity cDNA Reverse Transcription Kit (ThermoFisher Scientific) on a T100 Thermal Cycler (Bio-Rad). To evaluate the mRNA level of host genes including IL-6, CDK1, PLK1 and CCNA2, and viral genes including KSHV-encoded open reading frame (*ORF)73* (LANA), *ORF50* (regulator of transcription activator [RTA]), *K2* (viral interleukin-6 [vIL-6]), *vFLIP*, and *vCyclin*, SYBR green qPCR assays were performed using the Applied Biosystems^®^ SYBR^®^ Green PCR Master Mix (ThermoFisher Scientific) on an ABI StepOnePlus Real-time PCR system (ThermoFisher Scientific). Primers used are listed in Supplementary Fig. S1. Relative mRNA expression levels were analyzed using the ΔΔCt method with genes coding for β-actin as the reference gene.

### Western blot

Cells were plated at 3 × 10^5^ cells/mL for 48 h in the absence or presence of pacritinib. Whole cell lysates were prepared using M-PER mammalian protein extraction reagent (ThermoFisher Scientific, Waltham, MA) according to the manufacturer’s protocol. Lysates used for the analyses of phosphorylated STAT3, p65 and FLT3 were extracted in the presence of both protease inhibitor cocktail (ThermoFisher Scientific, Cat. No. 78429) and phosphatase inhibitor cocktail (ThermoFisher Scientific, Cat. No. 78420) at a final 1X concentration. Protein concentrations were determined using Pierce™ BCA Protein Assay Kit (ThermoFisher Scientific, Cat. No. 23225). Equal amounts of protein were run on 4 to 12% NuPAGE Bis–Tris precast gels (ThermoFisher Scientific, Cat. No. NP0322BOX), and transferred to a nitrocellulose membrane with iBlot (Life Technologies Grand Island, NY). Membranes were blocked overnight with 5% w/v BSA in Odyssey blocking buffer (Licor, Lincoln, NE). Primary antibodies used were: mouse anti-β-actin (Sigma, cat# A2228), rabbit anti-human JAK2 (Cell Signaling, cat# 3230S), rabbit anti-human pJAK2 (Cell Signaling, cat# 3771S), rabbit anti-human STAT3 (Cell Signaling, cat# 12640S), rabbit anti-human pSTAT3 (Cell Signaling, cat# 9145S), rabbit anti-human p65 (Cell Signaling, cat# 8242S), mouse anti-human pp65 (Cell Signaling, cat# 3043S), and rabbit anti-human FLT3 (Cell Signaling, cat# 3462S), rabbit anti-human pFLT3 (Cell Signaling, cat# 3463S). Secondary antibodies were conjugated to alkaline phosphatase (Promega, Madison. WI) or conjugated to green or red fluorescent dyes for use with LI-COR system. Blots were analyzed using the Odyssey imaging system and ImageStudio software (Li-Cor).

### Analysis of KSHV virus production by western blot

Cells were plated at 2 × 10^5^ cells per mL in RPMI medium with 5 nmol/L TPA, 0.2 μM pacritinib or DMSO control for 72 h. Supernatant was then collected and processed as described previously^51^. Concentrated virus was resuspended and subjected to Western blot analysis of ORF45, a tegument protein in the KSHV virion using an antibody against KSHV ORF45 (Abcam, ab36618).

### Statistical analysis

Statistical analysis was performed using paired two-sided t-tests on experiments with 3 or more biological replicates. P-values less or equal to 0.05 were considered statistically significant. Asterisks indicate p values: *p < 0.05, **p < 0.01, *** p < 0.001, **** p < 0.0001.

### Supplementary Information

Supplementary Information.

## Data Availability

The materials and data used during the current study are available from the corresponding author upon reasonable request. Sequencing data produced in this work have been submitted to SRA database with BioProject accession number PRJNA991521, and are available at: https://dataview.ncbi.nlm.nih.gov/object/PRJNA991521?reviewer=qa093hk7alq9r9e4ib9ssceakj.
