# Supplementary material for: Pacritinib inhibits proliferation of primary effusion lymphoma cells and production of viral interleukin-6 induced cytokines
Source: Sci Rep. 2024 Feb 19;14:4125. doi: 10.1038/s41598-024-54453-7 (PMC10876599; doi:10.1038/s41598-024-54453-7)
Supplement: Supplementary file 1 — Supplementary Information. [file 41598_2024_54453_MOESM1_ESM.pdf]

Pacritinib Inhibits Proliferation of Primary Effusion Lymphoma Cells and Production of Viral IL-6-induced Cytokines

Yiquan Wu, Victoria Wang, and Robert Yarchoan*

Supplemental Files

Figure S1 Primers used for RT-qPCR

Gene Name	Forward Primer Sequence (5' to 3')	Reverse Primer Sequence (5' to 3')
ORF73 (LANA)	CGCGAATACCGCTATGTACTCA	GGAACGCGCCTCATACGA
ORF50 (RTA)	TTGCCAAGTTTGTACAACTGCT	ACCTTGCAAAGACCATTCAGAT
K13 (vFLIP)	CGTCTACGTGGAGAACAGTGAGCT	CTGGGCACGGATGACAGGGAAGTG
ORF74 (vGPCR)	CAAGCAGGCCATGTGTTATG	AGCACCACAGCAACAATCAC
K2 (vIL6)	CTGTTACCGTACCGGCATCT	GGGTGGACTGTAGTGCGTCT
CDK1	TCGTGAACCAATCCCAGCCAAG	TCCACCCAGTTTGAGAAGCATC
IL6	CATCCTAGAGGACTCTGT	TTGCTCGTCTTCCAGTGT
HMGCR	GGACAGCCGTTGCCCTAGTGG	AGCGGACAAGCCGAATACCCTTC
PLK1	CGTGCCAATCTTGAGGTTTT	CGGAGGCGTGGTTAAATAAA
ACTB	CCTTCCTGGGCATGGAGT	CAGGGCAGTGATCTCCTTCT

Figure S2 Plasma serum Cmax concentrations of tested inhibitors

Drug Name	JAK Target (s)	Plasma Serum Cmax (μM)	Reference
AZD1480	JAK2	2.87	Plimacket al., 2013
Baricitinib	JAK1, JAK2	0.5	Shi et al., 2014
Pacritinib	JAK2	21	Singer et al., 2016
Peficitinib	JAK2	2.3	Miyatake et al., 2020
Ruxolitinib	JAK1, JAK2	7	Chen et al., 2014

Tested inhibitors and relative plasma serum Cmax concentrations are listed in the table.

Figure S3 Effect of pacritinib over time in PEL lines and lymphoma lines without KSHV

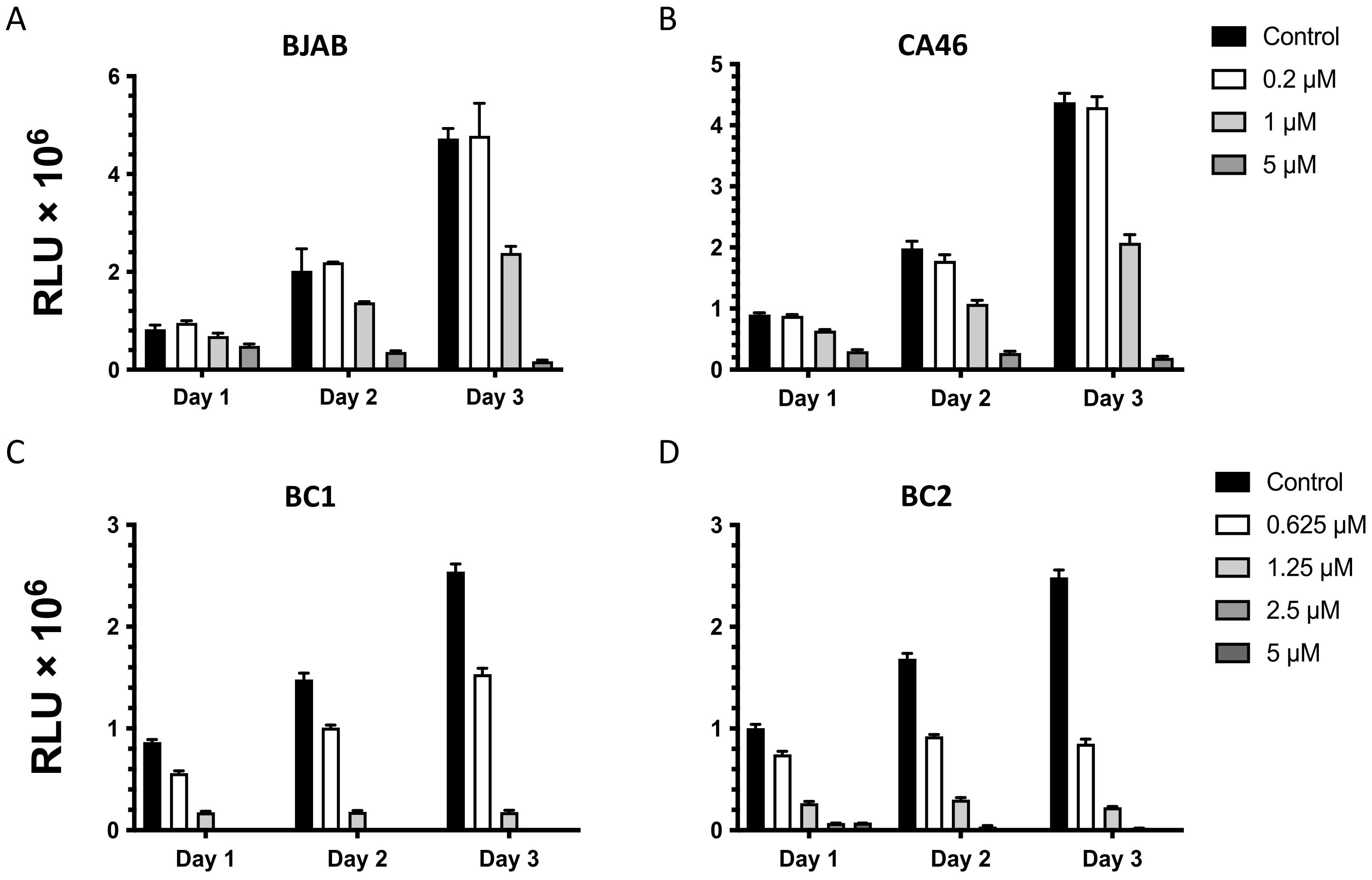

BJAB (A), CA46 (B), BC-1 (C), BC-2 (D) were treated in triplicate with indicated concentrations of pacritinib, or with RPMI medium control for 1, 2, and 3 days. The number of viable cells was assessed using CellTiter-Glo® Luminescent Cell Viability Assay. Shown is the data from one representative experiment from 3 independent experiments. Error bars indicate the standard deviations from 3 technical replicates.

Figure S4 Comparison of pacritinib from two sources in JSC-1 and BCBL-1

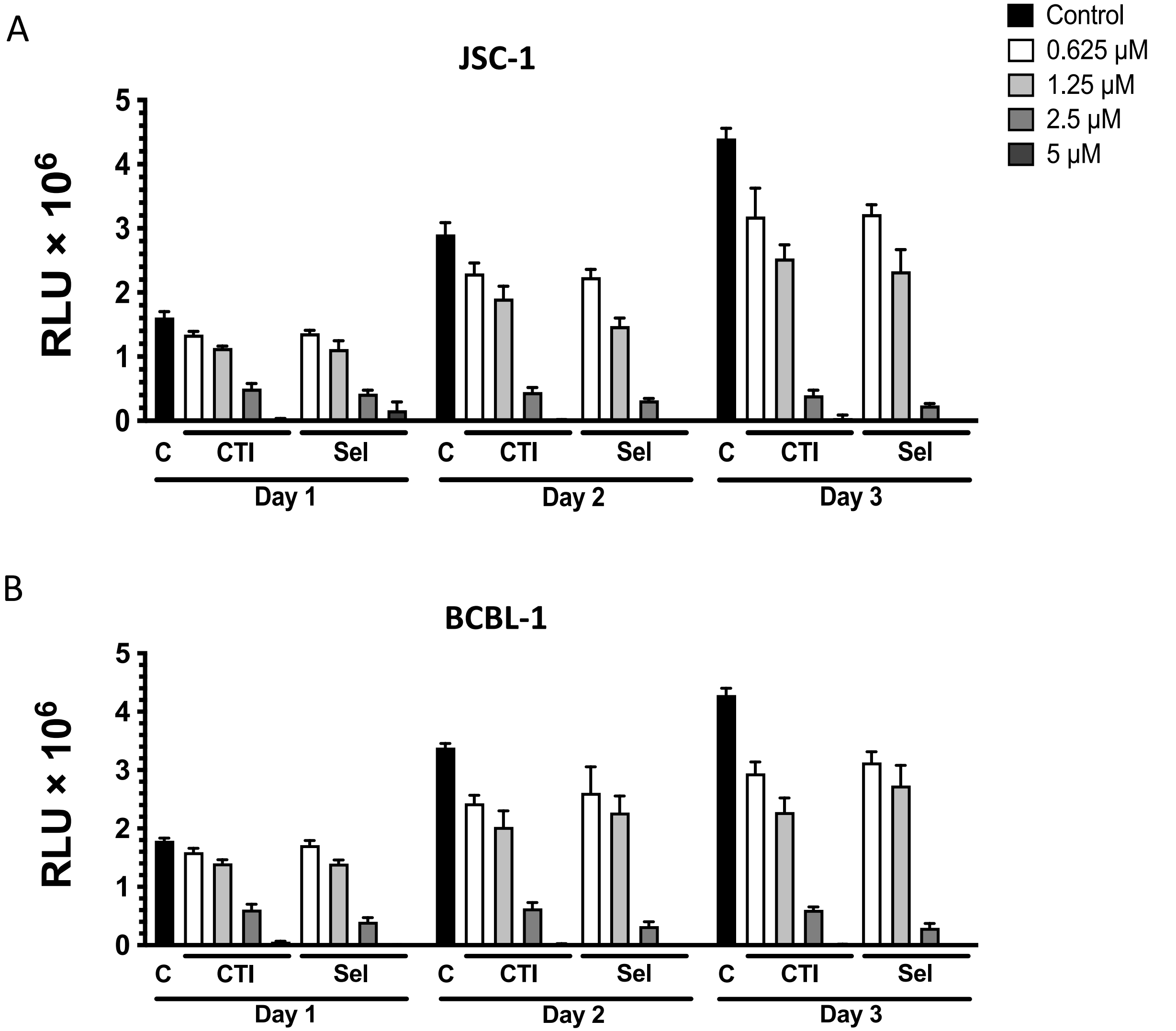

JSC-1 (A) and BCBL-1 (B) cells were treated in triplicate with indicated concentrations of pacritinib, or with RPMI medium control for 1, 2, and 3 days. The number of viable cells was assessed using CellTiter-Glo Luminescent Cell Viability Assay. Results shown are from 3 experiments. Error bars represent standard deviations from the 3 experiments. CTI and Sel represent pacritinib obtained from CTI BioPharma Corp. and Selleck Chemicals LLC respectively.

Figure S5 Flow cytometry analysis of pacritinib-induced cell apoptosis and cell death on cell lines with or without KSHV

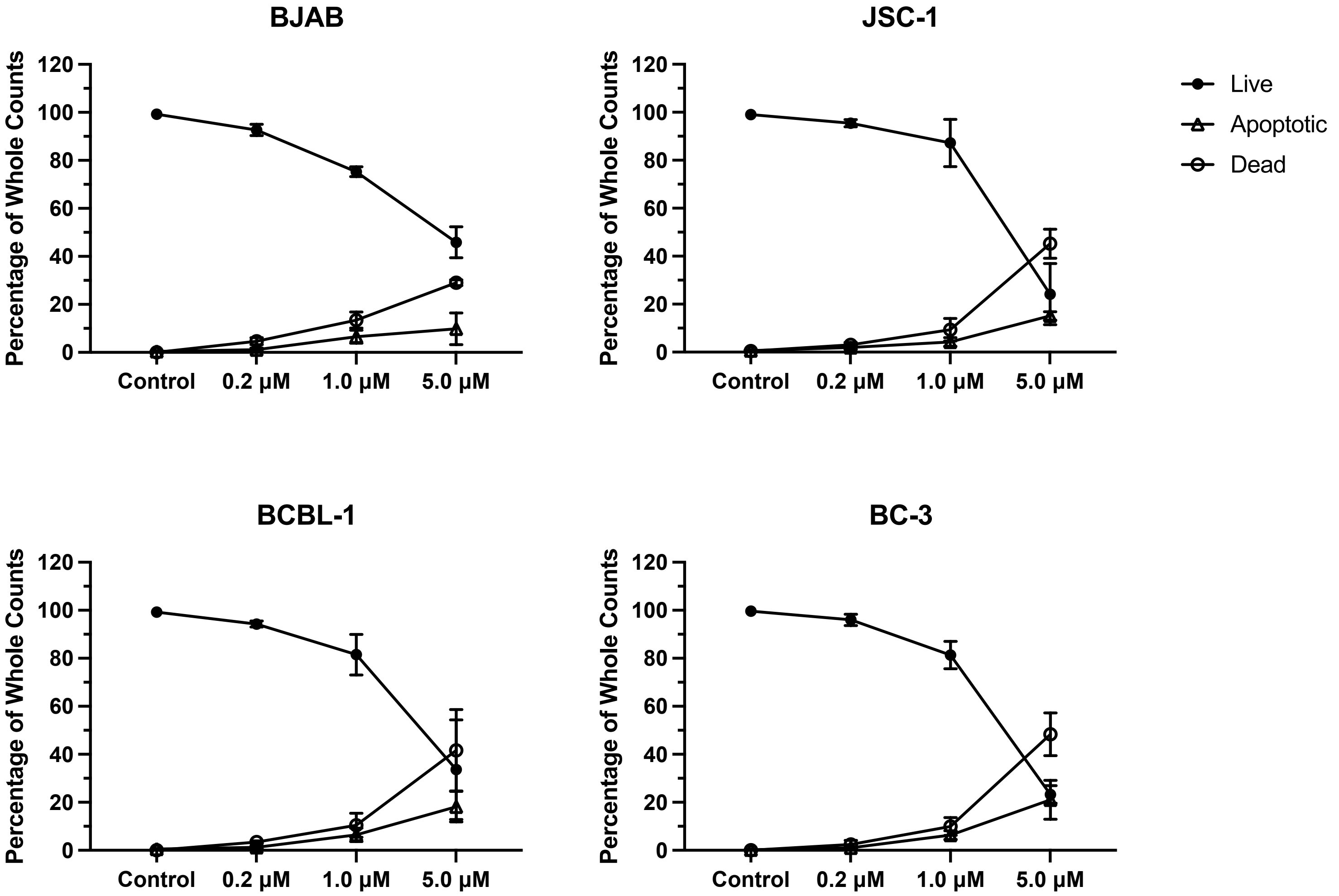

BJAB, JSC-1, BCBL-1, and BC-3 cells were treated for 24h with 0.2 μM, 1 μM, 5 μM pacritinib (Selleck Chemicals LLC) or DMSO control (Control), then stained with FITC Annexin V Apoptosis Detection Kit with 7-AAD. Cell apoptosis (Annexin V) and cell death (7-AAD) were analyzed by flow cytometry. Shown are the percentages of live, apoptotic, and dead cells relative to the whole population. Error bars represent standard deviations from the 3 experiments.

Figure S6 Effects of various FLT3 inhibitors on PEL cell growth

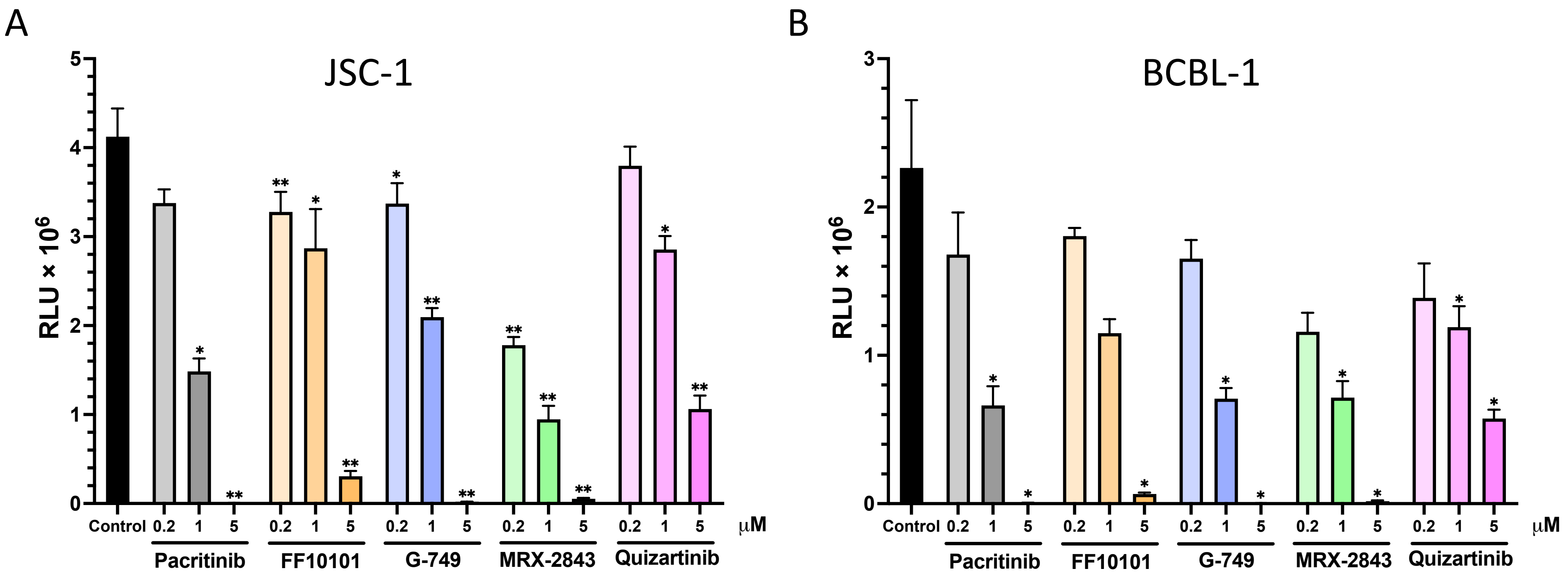

JSC-1 (A) and BCBL-1(B) cells were treated with indicated amount of pacritinib, FF10101, G-749, MRX-2843 or Quizartinib for 72h, and then the number of viable cells was assessed using CellTiter-Glo Luminescent Cell Viability Assay. RLU: relative luciferase unit. Results shown are from 3 experiments. Error bars represent standard deviations from the 3 experiments. Asterisks indicate p values: *p<0.05, **p<0.01 ***p<0.001, ****p<0.0001. Those without asterisks are not significant (p>0.05).

Figure S7 Growth inhibitory effect of pacritinib, gilteritinib and AZD1480 in JSC-1 and BCBL-1 cells

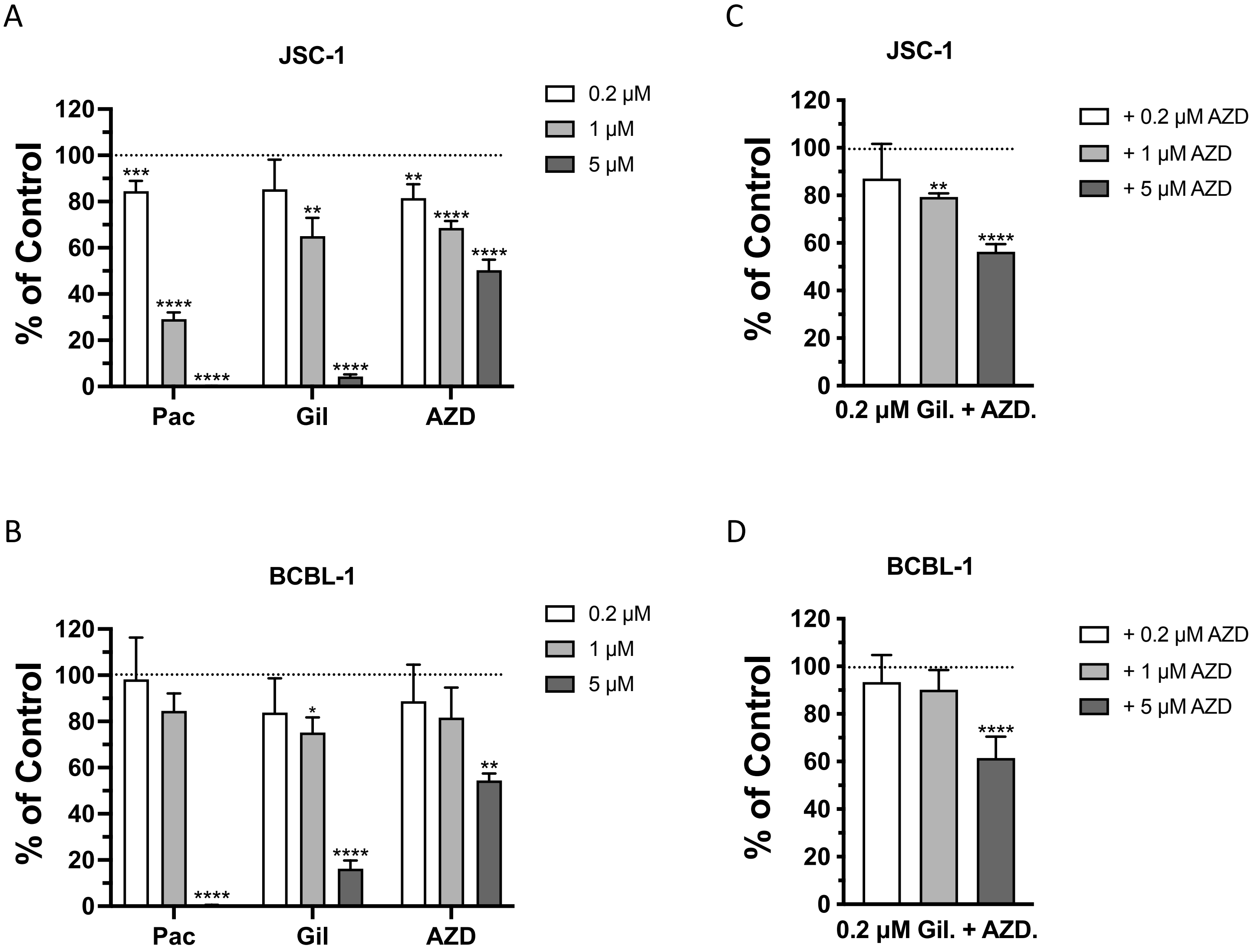

JSC-1 and BCBL-1 cells were treated for 72h with 0.2 μM, 1 μM, 5 μM pacritinib (Selleck Chemicals LLC), gilteritinib, AZD1480, DMSO control (Control) or combination of 0.2 μM gilteritinib with indicated amount of AZD1480, and then the number of viable cells was assessed using CellTiter-Glo Luminescent Cell Viability Assay. A: separate drugs in JSC-1 cells; B: separate drugs in BCBL-1 cells; C: combination of gilteritinib and AZD1480 in JSC-1 cells; D: combination of gilteritinib and AZD1480 in BCBL-1 cells. Shown are the percentages of viable cells relative to controls. Error bars represent standard deviations from the 3 experiments.

Figure S8 Co-deregulated genes in JSC-1 and BCBL-1 treated with pacritinib

A

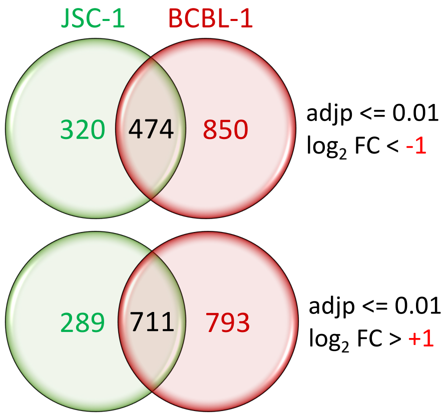

B

Co-up	Co-down
AKR1B10	KIF20A
DNAJC27-AS1	ESPL1
ABCA13	PLK1
SOST	CENPA
	SPC24
	CDCA3
	AC099329.1
	HIST2H3D
	CDC20P1
	ABCA1
	UBE2C
	SPC25
	NRGN
	HIST1H2AL
	HIST2H3C
	CCNA2
	HIST1H2BM
	HIST1H1T
	CDC20
	GTSE1
	HIST1H2AB
	HIST1H3G
	HIST1H1B
	KIF18B

* (|FC|>8, adjp<0.0001)

mRNA expression of cellular genes in JSC-1 and BCBL-1 cells treated for 24h with 0.5 µM pacritinib or DMSO control was measured by RNA-Seq. (A) Pie chart summarizing the overlapping status of DEGs in JSC-1 cells (green) and BCBL-1 cells (red). (B) Table lists most significant co-upregulated genes and co-downregulated genes in both JSC-1 and BCBL-1 groups.

Figure S9 Enriched pathways in pacritinib-treated JSC-1 and BCBL-1

Enriched pathways in JSC-1 group

Pathway	z-score	-log(p-value)
Salvage Pathways of Pyrimidine Ribonucleotides	-2.45	2.39
Cyclins and Cell Cycle Regulation	-2.24	2.07
Mitotic Roles of Polo-Like Kinase	-2.11	10.60
Cell Cycle Control of Chromosomal Replication	-2.00	1.95
Pyridoxal 5'-phosphate Salvage Pathway	-2.00	1.74
Role of BRCA1 in DNA Damage Response	-1.00	1.44
ATM Signaling	1.63	3.10
Cholesterol Biosynthesis	2.00	4.44
CHK Proteins in Cell Cycle	2.00	3.60
Cell Cycle: G2/M Regulation	2.12	6.09
NER Pathway	2.31	8.13
Superpathway of Cholesterol Biosynthesis	2.65	6.62

Enriched pathways in BCBL-1 group

Pathway	z-score	-log(p-value)
Cell Cycle Control of Chromosomal Replication	-2.65	3.12
Tetrakisphosphate Biosynthesis	-2.53	2.31
3-phosphoinositide Degradation	-2.53	2.02
D-myo-inositol-5-phosphate Metabolism	-2.53	2
Estrogen-mediated S-phase Entry	-2.45	4.25
Cyclins and Cell Cycle Regulation	-2.33	3.46
Superpathway of Inositol Phosphate Compounds	-2.31	2.11
Salvage Pathways of Pyrimidine Ribonucleotides	-2.12	2.31
3-phosphoinositide Biosynthesis	-2.11	2.28
Mitotic Roles of Polo-Like Kinase	-1.94	10.8
Pyridoxal 5'-phosphate Salvage Pathway	-1.63	2.08
Cell Cycle: G2/M Regulation	1.16	8.28
NER Pathway	1.27	3.95
Superpathway of Cholesterol Biosynthesis	2.24	3

DEGs from JSC-1 and BCBL-1 groups are separately submitted to IPA for pathway enrichment analysis. Enriched pathways with cut-off of $p < 0.05$ and $|Z\text{-score}| \geq 1$ were selected and listed in the tables.

Figure S10 Realtime PCR confirmation of human and KSHV genes expression in pacritinib-treated JSC-1 and BCBL-1

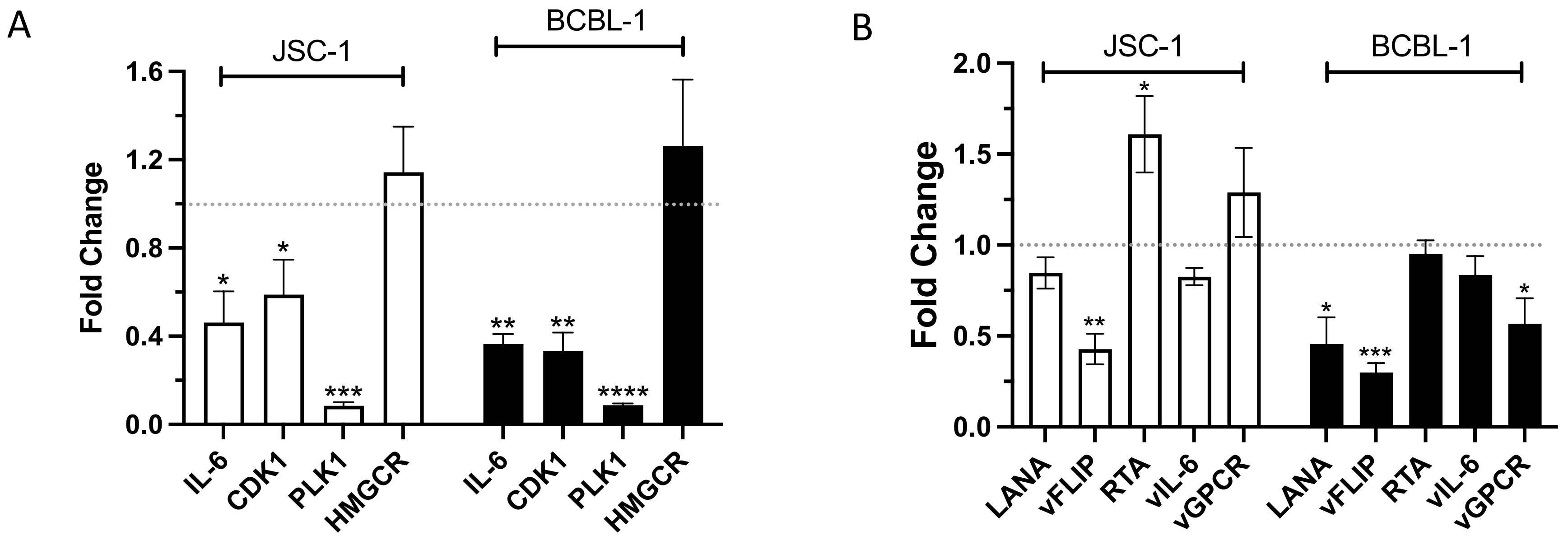

RNA extracted from JSC-1 and BCBL-1 cells treated for 24h with 0.5 μM pacritinib or DMSO control was subjected to qPCR for mRNA expression analysis of both human and KSHV genes. (A) Expression of human genes including IL-6, CDK1, PLK1 and HMGCR in JSC-1 and BCBL-1 cells were assessed using qPCR. Shown is the fold change of genes in pacritinib-treated cells compared in control cells from 3 independent experiments. (B) Expression of KSHV genes including LANA, vFLIP, RTA, vIL-6 and vGPCR in JSC-1 group and BCBL-1 group was assessed using qPCR. Shown is the fold change of genes in pacritinib-treated cells compared with genes in control cells from 3 independent experiments. Error bars indicate the standard deviations. Asterisks indicate p values: *p<0.05, ***p<0.001, ****p<0.0001. Those without asterisks are not significant (p>0.05).

Figure S11 Pacritinib induces mild production of KSHV virus in JSC-1 but not in BCBL-1

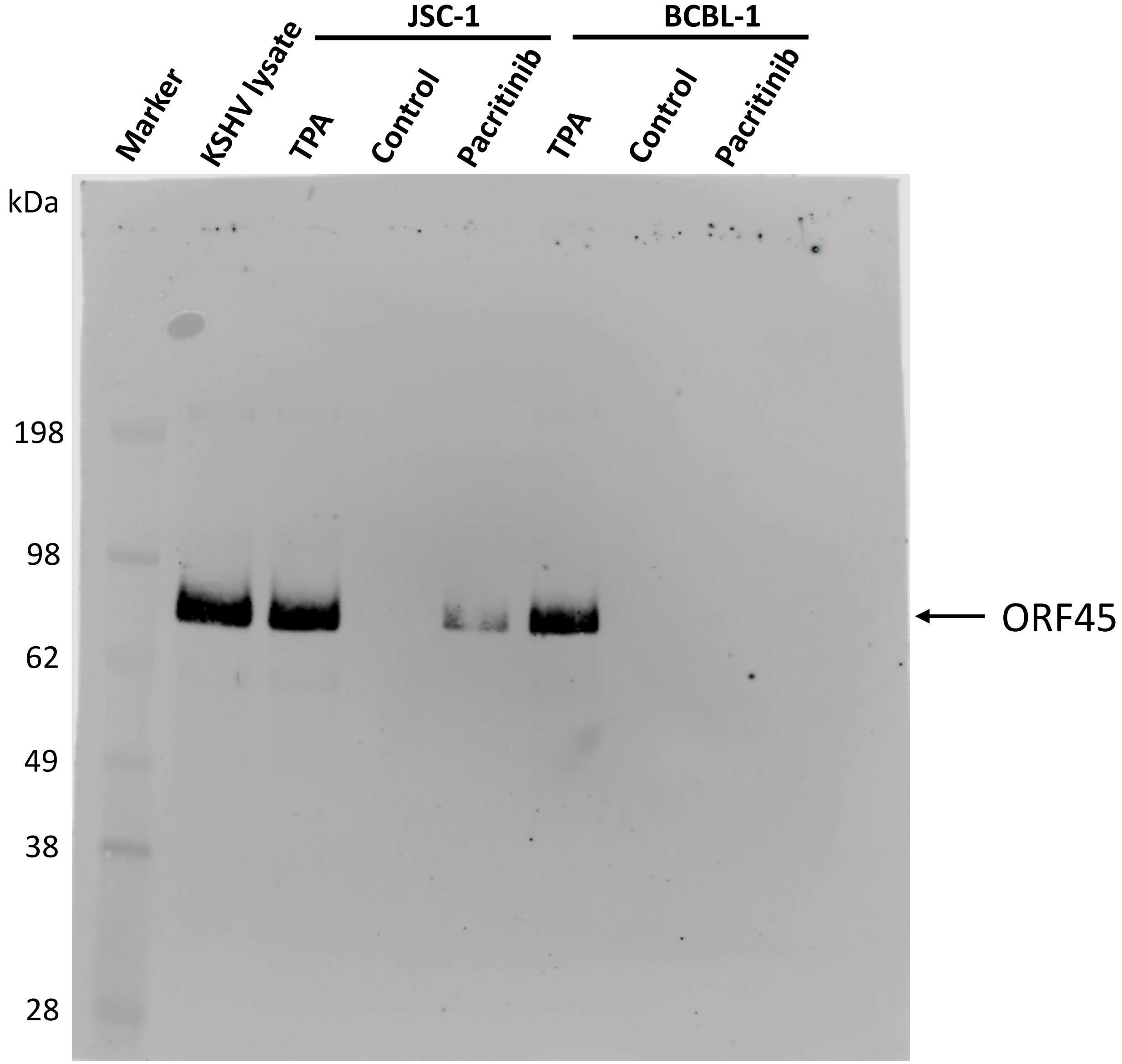

JSC-1 and BCBL-1 cells treated for 72h with 5 nmol/L TPA, 0.2 μM pacritinib or DMSO control. Supernatants were collected and ultracentrifuged to obtain viral pellets. The pellets were resuspended in SDS sample buffer and analyzed by Western blot using an antibody against a tegument protein of KSHV virion, ORF45 gene product.

Supplemental file 12 Full versions of all Western blots used in Figure 3A and Figure 5A

Figure 3A

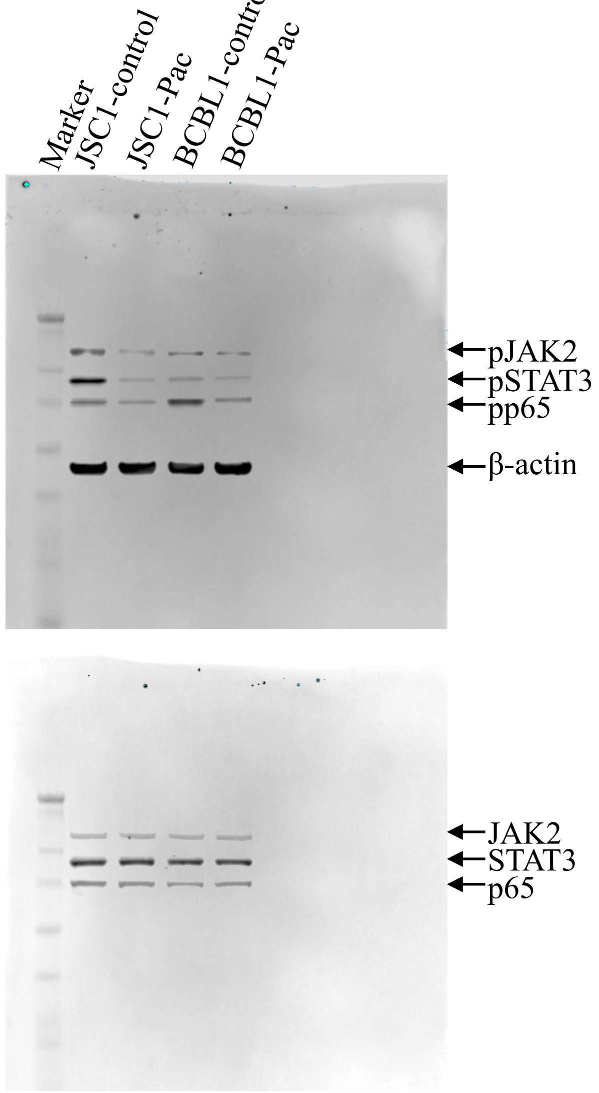

Figure 5A

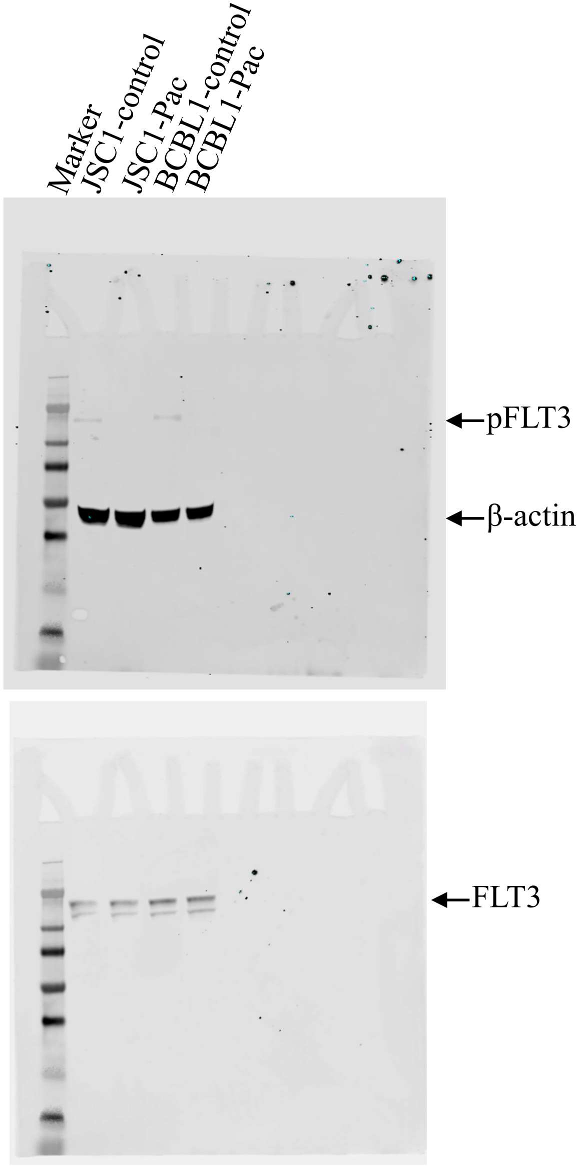

These full versions of Western blots images were exported using ImageStudio as original TIFF files after being scanned using the Odyssey Li-Cor.
